# Comparing Prevalence Estimates From Population-Based Surveys to Inform Surveillance Using Electronic Health Records

**DOI:** 10.5888/pcd14.160516

**Published:** 2017-06-08

**Authors:** Kathleen S. Tatem, Matthew L. Romo, Katharine H. McVeigh, Pui Ying Chan, Elizabeth Lurie-Moroni, Lorna E. Thorpe, Sharon E. Perlman

**Affiliations:** 1 New York City Department of Health and Mental Hygiene, Long Island City, New York; 2 City University of New York School of Public Health, New York, New York; 3 New York University School of Medicine, Department of Population Health, New York, New York

## Abstract

**Introduction:**

Electronic health record (EHR) systems provide an opportunity to use a novel data source for population health surveillance. Validation studies that compare prevalence estimates from EHRs and surveys most often use difference testing, which can, because of large sample sizes, lead to detection of significant differences that are not meaningful. We explored a novel application of the two one-sided *t* test (TOST) to assess the equivalence of prevalence estimates in 2 population-based surveys to inform margin selection for validating EHR-based surveillance prevalence estimates derived from large samples.

**Methods:**

We compared prevalence estimates of health indicators in the 2013 Community Health Survey (CHS) and the 2013–2014 New York City Health and Nutrition Examination Survey (NYC HANES) by using TOST, a 2-tailed *t* test, and other goodness-of-fit measures.

**Results:**

A ±5 percentage-point equivalence margin for a TOST performed well for most health indicators. For health indicators with a prevalence estimate of less than 10% (extreme obesity [CHS, 3.5%; NYC HANES, 5.1%] and serious psychological distress [CHS, 5.2%; NYC HANES, 4.8%]), a ±2.5 percentage-point margin was more consistent with other goodness-of-fit measures than the larger percentage-point margins.

**Conclusion:**

A TOST with a ±5 percentage-point margin was useful in establishing equivalence, but a ±2.5 percentage-point margin may be appropriate for health indicators with a prevalence estimate of less than 10%. Equivalence testing can guide future efforts to validate EHR data.

## Introduction

Electronic health records (EHRs) have generated enthusiasm for real-time population health surveillance, but understanding their comparability with other data sources, especially gold-standard sources, is crucial (1). A few studies have compared data from EHRs with data from surveys or registries by making hypothesis-testing statistical comparisons (2–9). With the exception of our recent EHR validation studies (7–9), these studies relied mostly on difference testing, which can establish only whether the difference between any 2 estimates is statistically significant. With difference testing, equivalence cannot be established, because a lack of a significant difference could simply result from insufficient power to detect a difference. Large sample sizes might also lead to the detection of significant differences that are not meaningful, which is a problem that other investigators have reported (2,5) and is cited as a reason to avoid the statistical testing of EHR data (10).

Because EHR data typically have large sample sizes, an alternate method of comparison, the two one-sided *t* test (TOST) or equivalence test, may be particularly helpful in comparing EHR data with data from other sources. Equivalence testing establishes that 2 estimates are statistically equivalent, which is conceptually distinct from establishing that 2 estimates are statistically different. TOST determines whether 2 estimates do not differ by more than a prespecified margin of equivalence, or equivalence margin (11). For TOST, the null hypothesis is that 2 estimates differ by more than the prespecified acceptable amount, allowing establishment of equivalence. For a *t* test, the null hypothesis is that 2 estimates are not different; therefore, even if the null hypothesis is accepted, a *t* test cannot establish equivalence.

The use of TOST is well established in the pharmaceutical industry for noninferiority trials, and both the US Food and Drug Administration and the European Medicines Agency provide guidelines for conducting equivalence testing and selecting equivalence margins (12,13). There is, however, no evidence-based precedent for establishing equivalence margins for prevalence estimates. In this study, we explored a novel application of TOST to compare prevalence estimates from 2 New York City population-based surveys in an effort to establish the optimal equivalence margin for validating prevalence estimates generated from EHR data.

## Methods

We used data from the 2013–2014 New York City Health and Nutrition Examination Survey (NYC HANES) and the 2013 Community Health Survey (CHS). NYC HANES is a population-based, cross-sectional household survey of noninstitutionalized New York City residents aged 20 years or older. The survey, modeled after the National Health and Nutrition Examination Survey, was conducted jointly by the City University of New York (CUNY) School of Public Health and the New York City Department of Health and Mental Hygiene (NYC DOHMH) (14). CHS is an annual cross-sectional, random-digit–dial telephone-based survey of New York City residents aged 18 years or older, modeled after the Behavioral Risk Factor Surveillance System (15). For both data sources, our analytic study population was restricted to adults aged 20 years or older who reported having seen a health care professional for primary care in the previous 12 months (“in care”) (16) and who had complete data for age, sex, and ZIP code. The in-care populations of the 2013 CHS (N = 6,166) and 2013–2014 NYC HANES (N = 1,135) were described previously (16) and had similar distributions in demographic characteristics, including age group, sex, race/ethnicity, education, and neighborhood poverty level. The protocol for the 2013 CHS was approved by the NYC DOHMH institutional review board, and the protocol for 2013–2014 NYC HANES was approved by the institutional review boards of both NYC DOHMH and the CUNY School of Public Health.

### Measures

We used data on the following 10 health indicators: smoking, influenza vaccination, depression, hypertension, diabetes, hyperlipidemia, serious psychological distress, and 3 categories of body mass index (BMI). Smoking was defined as having smoked at least 100 cigarettes in one’s lifetime and having recently smoked every day or some days at the time of the survey. Influenza vaccination was defined as reporting to have received an influenza vaccine in the previous 12 months. Depression, hypertension, diabetes, and hyperlipidemia were defined as an affirmative response to 4 questions asking respondents whether they had ever been told by a health care professional they had these conditions. The question on hyperlipidemia was restricted to men aged 40 years or older and women aged 45 years or older to be consistent with routine cholesterol testing recommendations of the US Preventive Services Task Force (17). Serious psychological distress was defined as a Kessler 6 score of at least 13 (of a possible 24) (18). BMI was classified into 3 categories: overweight or obesity (BMI ≥25), obesity (BMI ≥30), and extreme obesity (BMI ≥40). BMI was calculated as weight in kilograms divided by height in meters squared; height and weight were self-reported in CHS and measured during the interview in NYC HANES.

### Statistical analysis

We first generated prevalence estimates and 95% confidence intervals (CIs) using SAS-callable SUDAAN 11.0 (Research Triangle Institute) to account for the complex survey design. These estimates were weighted to the 2010 US census population (19), adjusted by using the 2008–2013 estimates from the American Community Survey (20), and age-standardized to the US 2000 standard population (21). For each pair of prevalence estimates for the 10 health indicators, we computed the absolute percentage-point difference in prevalence estimates and the prevalence ratio (using NYC HANES as the denominator). We conducted a 2-tailed *t* test and TOST in SAS 9.4 (SAS Institute Inc) using PROC TTEST; the TOST used the TOST option, and the 2-tailed *t* test did not use this option. We used population summary statistics (MEAN = adjusted population prevalence estimate, N = sample size, STD = adjusted standard deviation) computed in SUDAAN. The significance level was set at an α of .05.

For TOST, we hypothesized that a ±5 percentage-point margin would fit best for most health indicators on the basis of a previous study comparing vaccine coverage among various races in a national survey (22) and consultation with another jurisdiction using TOST to evaluate EHRs for surveillance (P. Joseph Gibson, Marion County Public Health Department, written and oral communications, 2016). We also tested a lower margin of ±2.5 percentage points and a higher margin of ±7.5 percentage points to assess whether margin size would vary according to prevalence magnitude. To determine the optimal equivalence margin for each health indicator, we compared TOST findings for each margin with 3 a priori goodness-of-fit criteria: a prevalence ratio of 0.85 to 1.15, an absolute difference in prevalence of 5 percentage points or less, and *t* test *P* ≥ .05.

## Results

In the comparison of CHS and NYC HANES prevalence estimates, 6 health indicators met all 3 goodness-of-fit criteria: influenza vaccination (CHS, 47.3%; NYC HANES, 47.6%), hyperlipidemia (CHS, 47.9%; NYC HANES, 46.9%), hypertension (CHS, 31.6%; NYC HANES, 32.5%), depression (CHS, 16.4%; NYC HANES, 15.2%), diabetes (CHS, 12.5%; NYC HANES, 12.6%), and serious psychological distress (CHS, 5.2%; NYC HANES, 4.8%) (Table). When we used TOST with a ±5 percentage-point margin, prevalence estimates from both surveys were statistically equivalent for influenza vaccination, hypertension, depression, diabetes, and serious psychological distress (*P* for all < .05), but not hyperlipidemia (*P* = .05). Smoking (CHS, 14.9%; NYC HANES, 17.7%) and extreme obesity (CHS, 3.5%; NYC HANES, 5.1%) met 2 of the 3 goodness-of-fit criteria. When we used TOST with a ±5 percentage-point margin, prevalence estimates from both surveys were not statistically equivalent for smoking (*P* = .09), but they were statistically equivalent for extreme obesity (*P* < .001). Overweight or obesity (CHS, 57.3%; NYC HANES, 65.9%) met only one of the 3 goodness-of-fit criteria, and when we used TOST with a ±5 percentage-point margin prevalence estimates from both surveys were not equivalent (*P* = .98). Obesity met none of the 3 goodness-of-fit criteria, and the prevalence estimates (CHS, 24.7%; NYC HANES, 31.3%) were not statistically equivalent when we used a ±5 percentage-point margin in TOST (*P* = .82).

**Table:** Prevalence Estimates, Measures of Difference, and Measures of Equivalence for Health Indicators in the In-Care Populations in the 2013 CHS and 2013–2014 NYC HANES, Ordered by Magnitude of Prevalence of Health Indicators

Health Indicator	% (95% CI)		Prevalence Ratio (CHS/NYC HANES)	Absolute Difference in Prevalence Between CHS and NYC HANES, Percentage Point (95% CI)^c^	*P* Value			
					*t* Test for Difference^d^	TOST for Equivalence, by Percentage-Point Margin^e^		
	CHS (N = 6,166)^a^	NYC HANES (N = 1,135)^b^				±5	±2.5	±7.5
Overweight or obesity^f^	57.3 (55.5 to 59.1)	65.9 (62.8 to 68.8)	0.87	−8.6 (−12.0 to −5.1)	<.001	.98	>.99	.73
Influenza vaccination^g^	47.3 (45.5 to 49.0)	47.6 (44.0 to 51.2)	0.99	−0.4 (−4.4 to 3.7)	.86	.01	.15	<.001
Hyperlipidemia^h^	47.9 (45.7 to 50.1)	46.9 (42.6 to 51.3)	1.02	0.9 (−4.0 to 5.8)	.71	.05	.26	.004
Hypertension^h^	31.6 (30.2 to 33.0)	32.5 (29.4 to 35.7)	0.97	−0.9 (−4.3 to 2.5)	.60	.01	.18	<.001
Obesity^f^	24.7 (23.2 to 26.3)	31.3 (28.5 to 34.2)	0.79	−6.5 (−9.8 to −3.3)	<.001	.82	.99	.28
Smoking^i^	14.9 (13.6 to 16.3)	17.7 (15.1 to 20.8)	0.84	−2.8 (−6.0 to 0.3)	.08	.09	.58	.002
Depression^h^	16.4 (15.1 to 17.9)	15.2 (13.0 to 17.7)	1.08	1.2 (−1.5 to 3.9)	.38	.003	.18	<.001
Diabetes^h^	12.5 (11.5 to 13.5)	12.6 (10.6 to 14.8)	0.99	−0.1 (−2.4 to 2.2)	.93	<.001	.02	<.001
Extreme obesity^f^	3.5 (2.9 to 4.2)	5.1 (3.8 to 6.8)	0.68	−1.6 (−3.2 to −0.02)	.05	<.001	.14	<.001
Serious psychological distress^j^	5.2 (4.5 to 6.1)	4.8 (3.5 to 6.5)	1.10	0.5 (−1.2 to 2.1)	.56	<.001	.009	<.001

Abbreviations: CHS, Community Health Survey; CI, confidence interval; NYC HANES, New York City Health and Nutrition Examination Survey; TOST, two one-sided *t* test.

a Weighted sample size is 4,137,212.

b Weighted sample size is 4,695,368.

c Value for CHS minus value for NYC HANES. Differences may vary by ±0.1 because of rounding.

d *P* value <.05 indicates that CHS and NYC HANES estimates were statistically different.

e *P* value <.05 indicates CHS and NYC HANES estimates were statistically equivalent.

f Body mass index (BMI) was classified into 3 categories: overweight or obesity (BMI ≥25), obesity (BMI ≥30), and extreme obesity (BMI ≥40). BMI was calculated as weight in kilograms divided by height in meters squared; height and weight were self-reported in CHS and measured during the interview in NYC HANES.

g Influenza vaccination was defined as reporting to have received an influenza vaccine in the previous 12 months.

h Depression, hypertension, diabetes, and hyperlipidemia were defined as an affirmative response to 4 questions asking respondents whether they had ever been told a by health care professional they had these conditions. The question on hyperlipidemia was restricted to men aged 40 years or older and women aged 45 years or older to be consistent with routine cholesterol testing recommendations of the US Preventive Services Task Force (17).

i Smoking was defined as having smoked at least 100 cigarettes in one’s lifetime and having recently smoked every day or some days at the time of the survey.

j Serious psychological distress was defined as a Kessler 6 score of at least 13 (of a possible 24) (18).

When we used a margin of ±2.5 percentage points, the prevalence estimates for diabetes (CHS, 12.5%; NYC HANES, 12.6%; *P* = .02) and serious psychological distress (CHS, 5.2%; NYC HANES, 4.8%; *P* = .009) were statistically equivalent (Figure) (Table). When we tested a margin of ±7.5 percentage points, the prevalence estimates for smoking (CHS, 14.9%; NYC HANES, 17.7%; *P* = .002) and hyperlipidemia (47.9% vs. NYC HANES, 46.9%; *P* = .004) were statistically equivalent. Only the prevalence estimates for overweight/obesity and obesity were not statistically equivalent at ±7.5 percentage points.

**Figure:**
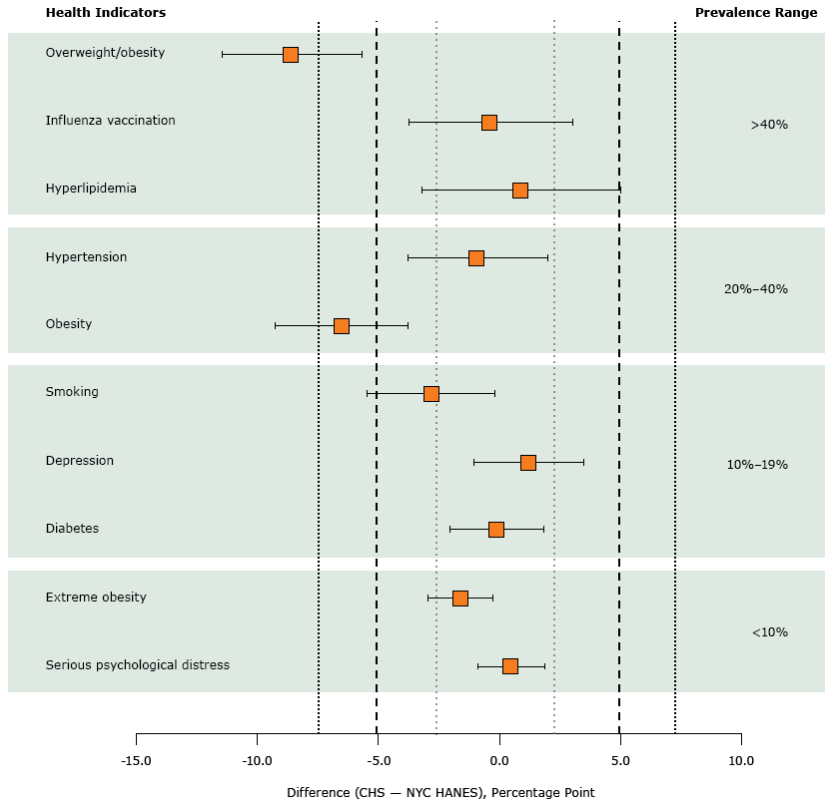
Prevalence estimates with 90% confidence intervals computed by using 3 TOST margins: ±2.5 percentage points (short dashed lines), ±5.0 percentage points (long dashed lines), and ±7.5 percentage points (medium-dashed lines). Health indicators are ordered in magnitude of prevalence in NYC HANES. Abbreviations: CHS, Community Health Survey; NYC HANES, New York City Health and Nutrition Examination Survey; TOST, two one-sided *t* test. **Table:** 

Health Indicator, by Prevalence Range	Percentage-Point Difference (90% Confidence Interval) in Prevalence (CHS – NYC HANES)
**Prevalence >40%**	
Overweight/obesity	−8.60 (−11.47 to −5.66)
Influenza vaccination	−0.40 (−3.73 to 3.01)
Hyperlipidemia	0.90 (−3.17 to 5.00)
**Prevalence 20%–40%**	
Hypertension	−0.90 (−3.78 to 1.97)
Obesity	−6.50 (−9.26 to −3.81)
**Prevalence 10%–19%**	
Current smoking	−2.80 (−5.47 to −0.21)
Depression	1.20 (−1.05 to 3.48)
Diabetes	−0.10 (−2.02 to 1.83)
**Prevalence <10%**	
Extreme obesity	−1.60 (−2.95 to −0.27)
Serious psychological distress	0.50 (−0.90 to 1.88)

## Discussion

In this analysis comparing prevalence estimates for health indicators between the in-care populations of 2013–14 NYC HANES and 2013 CHS, using TOST with a ±5 percentage-point margin was most appropriate for health indicators that had prevalence estimates ranging from 10% to almost 50% (eg, influenza vaccination). Among the various methods used in this study, only TOST allowed us to establish equivalence. TOST could play an important role in validating EHR data because it not only allows the assessment of equivalence but it also avoids the potential pitfalls of the *t* test. Prevalence ratio and absolute percentage-point difference in prevalence have additional shortcomings. Prevalence ratio is sensitive to the magnitude of the prevalence estimates and therefore cannot be reliably used to assess equivalence or difference across a wide range of estimates. Absolute percentage-point difference in prevalence, in contrast to prevalence ratio, is insensitive to the magnitude of prevalence estimates and could give a wrong sense of equivalence or difference.

We observed a better fit into the ±2.5 percentage-point margin as prevalence magnitude and standard errors decreased. Consistent with this pattern, a ±2.5 percentage-point margin for TOST appeared to be most appropriate for health indicators that had a prevalence estimate of less than 10%. For serious psychological distress, a ±2.5 percentage-point margin seemed most appropriate because the TOST result was significant and consistent with the other goodness-of-fit criteria, indicating that prevalence estimates were similar. Extreme obesity also had a low prevalence in both surveys, and the low prevalence ratio of 0.68 and *P* value of .05 indicated that the prevalence estimates were different. The prevalence estimates were statistically equivalent at the ±5 percentage-point margin but not at the 2.5 percentage-point margin. Therefore, it seems that a ±2.5 percentage-point margin would be most consistent with other criteria showing that the prevalence estimates were indeed not similar.

We have 3 recommendations for using TOST to assess the equivalence of prevalence estimates. First, we recommend that TOST margins be selected according to the public health importance of the difference in prevalence estimates, in line with drug regulatory agencies’ recommendation that margin selection should be guided by clinical relevance (12,13). For example, a ±5 percentage-point margin could be used for obesity because the estimates were clearly different and TOST using this margin demonstrated lack of equivalence. Self-reported height and weight, as recorded in CHS, is considered an acceptable way to measure obesity for public health surveillance, although we should expect to see differences when we compare data on self-reported height and weight in CHS with data on measured height and weight in NYC HANES. Second, we recommend that the size of the standard error of the difference in prevalence estimates be used to guide margin selection (ie, a small standard error calls for a smaller margin, and a large standard error calls for a larger margin). Because the size of the standard error depends on the sample size of the data sources and the prevalence of the health indicator, smaller margins may be needed when comparing surveys with large sample sizes and when prevalence estimates are small. Third, the type of data being compared should also inform margin selection. In public health surveillance, we usually are interested in estimates of prevalence and incidence (ie, proportions), but sometimes we are interested in comparing means. An important issue in comparing means is that a ±5 percentage-point margin has different clinical meanings for health indicators measured on different scales, such as BMI and hemoglobin A1_c_. Using standardized effect size of a relative percentage-point difference in estimates as a proxy for acceptable magnitude of difference might be useful for comparing means (23).

A strength of this study was the ability to compare the prevalence estimates for the same health indicators in 2 representative surveys from the same geographic area during the same time period. One limitation is the greater degree of imprecision (ie, wider confidence intervals) for some health indicators (eg, hyperlipidemia) compared with others (eg, hypertension). Although the greater degree of imprecision complicated margin selection when we examined individual health indicators, our choice of an optimal margin of ±5 percentage points was ultimately based on what was best for most indicators.

Equivalence testing may be a useful method for assessing similarity between EHR-based prevalence estimates and survey-based prevalence estimates. The NYC DOHMH, in collaboration with the CUNY School of Public Health, developed the NYC Macroscope, a primary care EHR-based surveillance system aimed at monitoring chronic conditions and risk factors (24). The ±5 percentage-point equivalence margin used in this study was used for NYC Macroscope validation studies (7–9) and could help guide future work in other jurisdictions. Although the use of a ±5 percentage-point margin was appropriate for most estimates, future research is needed to further define best practices for margin selection when validating EHR data.
